# Green-synthesized Fe-doped TiO_2_/ZnO heterojunction nanophotocatalyst for efficient visible-light-driven gas-phase degradation of toluene

**DOI:** 10.1039/d6ra03648a

**Published:** 2026-07-13

**Authors:** Reza Jamshidi, Amir Mirshafiee, Hamed Akbari, Mohammad Ali Amani, Mehdi Salari

**Affiliations:** a Student Research Committee, Baqiyatallah University of Medical Sciences Tehran Iran; b Health Research Center, Life Style Institute, Baqiyatallah University of Medical Sciences Tehran Iran a_mirsh1@yahoo.com; c Department of Environmental Health Engineering, Faculty of Health, Baqiyatallah University of Medical Sciences Tehran Iran; d Applied Biotechnology Research Center, New Health Technologies Institute, Baqiyatallah University of Medical Science Tehran Iran; e Non-Communicable Diseases Research Center, Sabzevar University of Medical Sciences Sabzevar Iran; f Department of Environmental Health Engineering, School of Public Health, Sabzevar University of Medical Sciences Sabzevar Iran amirmirshafiee14@gmail.com

## Abstract

In this study, a green-synthesized Fe-doped TiO_2_/ZnO heterojunction nanophotocatalyst was successfully fabricated *via* a combined sol–gel/hydrothermal method using pomegranate peel extract as a natural reducing and capping agent. The optimized catalyst (TiO_2_/ZnO = 70 : 30 w/w, 3 mol% Fe, 50 mL extract) exhibited a mesoporous structure with a high surface area (85 m^2^ g^−1^), reduced crystallite size (∼18 nm), and enhanced visible-light absorption due to Fe^3+^-induced defect states and oxygen vacancies. Photocatalytic performance was evaluated for gas-phase toluene degradation in a continuous-flow quartz reactor under visible LED irradiation (420–700 nm, 30 mW cm^−2^). The optimized sample (TZFA1) achieved 84% removal efficiency at 50 ppm within 120 min, following pseudo-first-order kinetics (*k*_app_ ≈ 0.014 min^−1^, *R*^2^ > 0.98). Reactive oxygen species trapping tests showed that O_2_^−^˙ plays the dominant role in toluene degradation, with additional contributions from ˙OH and h^+^, supporting the proposed photocatalytic mechanism. The enhanced performance is attributed to the synergistic effects of efficient charge separation *via* type-II TiO_2_/ZnO heterojunction, Fe^3+^/Fe^2+^ redox cycling, and improved surface properties induced by green modification. Systematic optimization of compositional and operational parameters confirmed the robustness of the system under varying conditions. The catalyst also demonstrated acceptable reusability over five cycles with moderate activity loss. These findings highlight a sustainable and efficient platform for practical VOC removal in air purification applications.

## Abstract 2

In this study, a green-synthesized Fe-doped TiO_2_/ZnO heterojunction nanophotocatalyst was successfully fabricated *via* a combined sol–gel/hydrothermal method using pomegranate peel extract as a natural reducing and capping agent.

## Graphical abstract

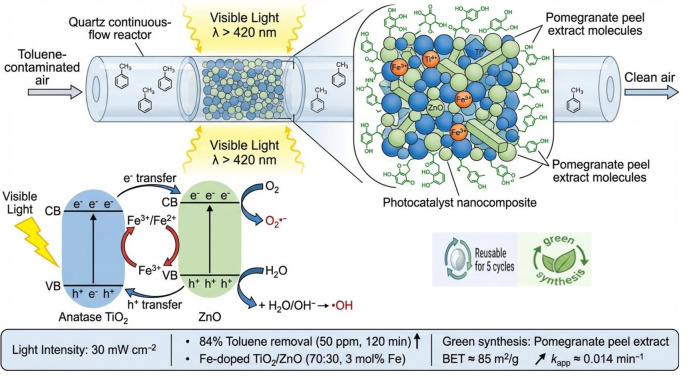

## 1. Introduction

Volatile organic compounds (VOCs) emitted from industrial activities, including military, chemical, and petrochemical operations, are major air pollutants that contribute to atmospheric photochemical reactions and secondary pollutant formation. Owing to their systemic toxicity and neurological effects, VOCs pose serious risks to environmental quality and human health.^1,2^ Toluene (C_7_H_8_), a prominent Benzene, Toluene, Ethylbenzene, and Xylenes (BTEX) component, is widely used in paints, resins, adhesives, explosives (TNT precursor), and aviation fuel octane boosters, contributing to secondary pollutant formation and ambient air contamination.^3–6^ Toluene exhibits a relatively high vapor pressure (28.4 mmHg at 25 °C) and low water solubility (∼0.47 g L^−1^), physicochemical properties well documented in authoritative sources such as NIST and EPA reports. These characteristics enhance its persistence in the gas phase—particularly in indoor or poorly ventilated environments—and make it a challenging volatile organic compound for photocatalytic removal.^7,8^

Inhalation of toluene causes respiratory irritation, neurotoxicity, and central nervous system depression, with exposure limits set by Occupational Safety and Health Administration-OSHA (PEL 200 ppm TWA, STEL 500 ppm), National Institute for Occupational Safety and Health-NIOSH (100 ppm TWA, 150 ppm STEL), and Environmental Protection Agency-EPA (MCL 1 mg L^−1^ in drinking water).^9,10^ These regulatory thresholds highlight the necessity of efficient control technologies for occupational and environmental exposure mitigation. Conventional removal technologies (absorption, adsorption, biodegradation, catalytic oxidation, plasma) often transfer pollutants rather than mineralize them, incurring high costs, secondary pollution, and low efficiency at dilute concentrations.^11–13^ Particularly in gas-phase systems with low VOC concentrations, incomplete oxidation and catalyst deactivation remain major operational challenges. Advanced oxidation processes (AOPs), particularly heterogeneous photocatalysis using semiconductor materials, offer a destructive pathway for VOC mineralization *via* reactive oxygen species (˙OH, SO_4_˙^−^, O_2_˙^−^) under mild conditions.^14,15^ Among various photocatalysts, TiO_2_ has long been considered a benchmark material owing to its excellent chemical stability, low toxicity, and high oxidative potential. Nevertheless, the practical application of pristine TiO_2_ is severely limited by its wide bandgap (∼3.2 eV), which restricts activation to ultraviolet light, as well as rapid recombination of photogenerated electron–hole pairs. To overcome these limitations, strategies such as constructing TiO_2_/ZnO heterojunctions and introducing transition metal dopants (*e.g.*, Fe) have been widely explored to enhance charge separation and extend light absorption into the visible region.^16,17^ Green synthesis using plant extracts (*e.g.*, pomegranate peel) provides eco-friendly routes.^18,19^ Furthermore, the use of visible LED irradiation instead of conventional mercury UV lamps offers advantages in energy efficiency, operational safety, lower heat generation, and environmental sustainability.^20–22^ In addition to traditional metal oxide semiconductors, other materials such as Metal–Organic Frameworks (MOFs) (*e.g.*, NH_2_-MIL-125(Ti) and UiO-66) and biomass-derived photocatalysts have also been explored for photocatalytic toluene degradation. However, many of these materials suffer from limitations including poor stability under humid conditions, complex synthesis routes, and insufficient reusability in gas-phase systems.^23,24^ In this context, the green-synthesized Fe-doped TiO_2_/ZnO heterojunction developed in this study offers a balanced and practical alternative, combining sustainability, visible-light responsiveness, and good operational stability.

Recent studies have demonstrated the efficacy of Fe-doped TiO_2_/ZnO composites. For instance, a removal efficiency of ∼79% was achieved under visible light for dye pollutants,^17^ and enhanced photocatalytic activity has been reported for green-synthesized Fe-doped ZnO/TiO_2_ in textile dye degradation.^25^ Despite these advancements, most reported studies focus on aqueous-phase pollutant degradation, while gas-phase VOC removal under visible-light irradiation remains insufficiently explored.^26^ Recent advances suggest that heterojunction engineering and transition-metal doping are effective approaches for improving photocatalytic performance through enhanced charge separation and electronic-structure modulation. In parallel, reactive oxygen species (ROS) identification provides key mechanistic insight into pollutant oxidation pathways. These aspects motivate the integration of Fe-doped TiO_2_/ZnO heterojunctions with ROS analysis for visible-light-driven gas-phase toluene degradation.^27–29^ Although TiO_2_/ZnO heterojunctions and Fe-doped oxide photocatalysts have been widely investigated, most studies have employed conventional chemical or hydrothermal synthesis methods and have primarily targeted aqueous-phase pollutants. In this context, the integrated use of Fe doping, TiO_2_/ZnO heterojunction engineering, green synthesis, and ROS-based mechanistic evaluation for visible-light-driven gas-phase toluene degradation remains insufficiently explored.^26,30^ To bridge existing gaps in visible-light-driven photocatalysis, this study reports the synergistic integration of heterojunction engineering and green synthesis. A novel Fe-doped TiO_2_/ZnO nanocomposite was fabricated using pomegranate peel extract as a sustainable capping agent, optimized specifically for the gas-phase degradation of toluene under energy-efficient visible LED irradiation. In contrast, gas-phase VOC removal under visible-light irradiation—particularly through the integrated use of Fe doping, heterojunction engineering, and green synthesis strategies—remains relatively underexplored.^31^

In this study, a synergistic and systematically integrated approach is introduced by combining Fe^3+^-induced defect engineering, TiO_2_/ZnO proposed type-II-like heterojunction construction, and green surface modification using pomegranate peel extract to fabricate a photocatalyst specifically optimized for gas-phase toluene degradation under visible LED irradiation. The resulting material exhibits a synergistic enhancement in photocatalytic activity that cannot be explained by the additive contributions of its individual components. Beyond material synthesis, this work provides a quantitative evaluation of the interplay between structural parameters (TiO_2_/ZnO ratio, Fe content, and extract loading) and operational conditions (light intensity and VOC concentration), offering new mechanistic insights into charge separation, interfacial electron transfer, reactive oxygen species generation, and overall photocatalytic efficiency in gas-phase systems.

The main objectives of this study were: (i) to synthesize and characterize the green-modified Fe-doped TiO_2_/ZnO nanocomposite, (ii) to evaluate its gas-phase photocatalytic performance for toluene degradation under visible LED irradiation in a continuous-flow reactor, and (iii) to optimize compositional and operational parameters influencing degradation efficiency and kinetics. Although several studies have investigated Fe-doped TiO_2_/ZnO composites or green-synthesized photocatalysts, the majority are limited to aqueous-phase pollutant degradation. To the best of our knowledge, the synergistic integration of green synthesis using pomegranate peel extract, Fe doping, and TiO_2_/ZnO heterojunction engineering specifically optimized for gas-phase toluene degradation under visible LED irradiation has not been systematically reported. The present work addresses this gap by developing a sustainable nanophotocatalyst with enhanced visible-light activity, high surface area, and acceptable reusability for practical VOC removal applications.

## 2. Materials and methods

### 2.1. Chemicals and reagents

Titanium(iv) isopropoxide (TTIP, Ti[OCH(CH_3_)_2_]_4_, 97%), zinc acetate dihydrate (Zn(CH_3_COO)_2_·2H_2_O, ≥98%), and iron(iii) nitrate nonahydrate (Fe(NO_3_)_3_·9H_2_O, ≥98%) were used as metal precursors and purchased from Merck (Germany). Absolute ethanol (99.9%), glacial acetic acid (100%), and hydrochloric acid (37%) were obtained from Merck and used as solvent and pH adjuster, respectively. Analytical-grade toluene (C_7_H_8_, ≥99.9%) was supplied by Merck. Fresh pomegranate peels were collected from local markets, thoroughly washed, air-dried at room temperature, and ground into fine powder before to use. All chemicals were of analytical grade and used without further purification. Deionized water (resistivity ≥18 MΩ cm) was used throughout all experiments.

### 2.2. Preparation of pomegranate peel extract

The pomegranate peel extract was prepared by a simple decoction method. Briefly, 10 g of dried and powdered pomegranate peel was added to 100 mL of distilled water and boiled for 10 min. The mixture was allowed to steep at room temperature for 24 h, then filtered through Whatman No. 1 paper. The clear filtrate was stored at 4 °C until use. This extract is rich in polyphenols (ellagic acid, punicalagin, *etc.*) that serve as both reducing and capping agents in the green modification step.^16,18,19,25^

### 2.3. Synthesis of Fe-doped TiO_2_/ZnO nanophotocatalyst

Fe-doped TiO_2_/ZnO nanoparticles were synthesized using a sol–gel method followed by hydrothermal treatment and calcination.^19,25^ Specifically, 10 mL of TTIP was dissolved in 40 mL of absolute ethanol to form solution A under continuous magnetic stirring (500 rpm). Solution B was prepared by dissolving the required amount of zinc acetate dihydrate (corresponding to TiO_2_ : ZnO weight ratios of 70 : 30, 50 : 50, and 30 : 70) in 30 mL of distilled water and 30 mL of ethanol, followed by the addition of 5 mL of glacial acetic acid as a stabilizing agent. The hydrolysis molar ratio (H_2_O : TTIP) was maintained at approximately 4 : 1 to ensure controlled sol–gel transition. The pH was adjusted to 2.0–3.0 using 1 M HCl under vigorous stirring. Solution C, containing Fe (NO_3_)_3_·9H_2_O dissolved in ethanol (1, 3, and 5 mol% Fe relative to total Ti + Zn, selected based on typical optimal doping levels for visible-light activity without phase segregation), was added to solution B under vigorous stirring. The Fe-containing solution B was then added dropwise to solution A under continuous stirring. The resulting sol was stirred for 2 h and aged for 24 h at room temperature to promote gel formation. The resulting gel was subjected to hydrothermal treatment at 100 °C for 2 h, followed by drying and calcination at 700 °C (heating rate: 3 °C min^−1^) to obtain crystalline nanoparticles. Although anatase-to-rutile transformation typically occurs above 600–700 °C, the presence of ZnO and Fe dopants can retard phase transition and stabilize anatase domains, as confirmed by XRD analysis in this study.^17^ The high calcination temperature was selected to enhance crystallinity and heterojunction formation while maintaining dominant anatase characteristics.

### 2.4. Green surface modification

0.5 g of the calcined Fe-doped TiO_2_/ZnO powder was dispersed in 25, 50, or 100 mL of pomegranate peel extract and stirred at 75 °C for 2 h. This step allows phytochemical constituents of pomegranate peel extract to participate in nanoparticle nucleation and growth, contributing to morphology control, inhibition of particle agglomeration, and defect generation during the synthesis process. The suspension was centrifuged (5000 rpm, 15 min), washed three times with distilled water and ethanol, and dried at 80 °C for 12 h.^18,19,25^

### 2.5. Characterization

Phase structure was analyzed by X-ray diffraction (XRD, Bruker D8 ADVANCE, Cu Kα, 10–80°). Morphology and particle size were examined by field-emission scanning electron microscopy (FE-SEM) at 15 kV. Elemental composition and mapping were obtained by energy-dispersive X-ray spectroscopy (EDX). Textural properties (BET surface area and BJH pore size distribution) were measured by N_2_ adsorption–desorption at 77 K (BELSORP MINI II).^17^

### 2.6. Experimental setup

Experiments were conducted in a continuous-flow cylindrical quartz reactor (inner diameter 8.3 cm, length 45 cm, volume ≈2.4 L). The catalyst (0.5 g) was coated on aluminum foil (26 × 45 cm) and inserted inside the reactor. A visible LED lamp (400–700 nm, intensity 5–30 mW cm^−2^) was used. The distance between the light source and the catalyst surface was maintained at a fixed position (15 cm). Toluene vapor was generated by a dynamic dilution system, and the total flow rate was fixed at 0.5 L min^−1^. Inlet and outlet concentrations were monitored in real time with a calibrated PhoCheck Tiger PID analyzer and periodically verified by GC-FID (Table 1).^15,32–34^

**Table 1:** The physicochemical properties of toluene

Structure	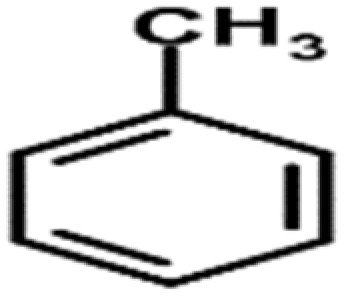
Formula	C_7_H_8_
Molar mass	92.1381 g mol^−1^
Density	0.8669 g mL^−1^ (20 °C)
Solubility	0.47 g L^−1^ (20–25 °C)
Viscosity	0.590 cP (20 °C)
Vapor pressure	28.4 mmHg (25 °C)

### 2.7. Experimental design and optimization

The effects of key operational parameters on toluene removal efficiency were investigated using a one-factor-at-a-time (OFAT) approach. The OFAT approach was employed for preliminary optimization due to its simplicity and effectiveness in identifying dominant parameters before advanced multivariate optimization methods.^25,35,36^ The parameters and their levels are summarized in Table 2. Experimental data obtained from the photocatalytic degradation of toluene were analyzed using descriptive and inferential statistical methods. All experiments were conducted in triplicate, and the results are reported as mean values ± standard deviation (SD) to ensure reproducibility and reliability of the data. The toluene removal efficiency (%) was calculated using the following eqn (1):1
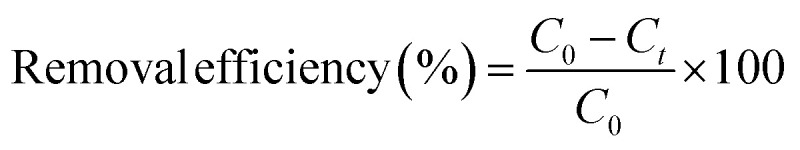
where *C*_0_ and *C*_*t*_ represent the initial and residual concentrations of toluene (ppm), respectively. The significance of observed variations was assessed using one-way analysis of variance (ANOVA), with a confidence level of 95% (*p* < 0.05).

**Table 2:** Investigated operational parameters and their levels

Parameter	Level				Unit
	i	ii	iii	iv	
Tio_2_/Zno ratio	70 : 30	50 : 50	30 : 70	—	%
Fe doping	1	3	5	—	%
Pomegranate peel extract volume	25	50	100	—	mL
Toluene concentration	50	200	500	1000	ppm
Reaction time	15	30	60	120	min
Light intensity	5	10	20	30	mw cm^−2^

### 2.8. Reactive oxygen species (ROS) scavenger experiments

To identify the dominant reactive oxygen species involved in the photocatalytic degradation of toluene, scavenger experiments were conducted under identical conditions to the standard photocatalytic tests. Benzoquinone (BQ, 1 mM), isopropanol (IPA, 1 mM), and ethylenediaminetetraacetic acid disodium salt (EDTA-2Na, 1 mM) were employed as scavengers for superoxide radicals (O_2_^−^˙), hydroxyl radicals (˙OH), and photogenerated holes (h^+^), respectively. Scavenger experiments were performed by adding 1 mM of each scavenger directly into the gas-phase reactor or by pre-treating the catalyst film with scavenger solution (1 mM in ethanol/water) followed by drying at 80 °C. Control experiments without scavenger were run in parallel. All tests were conducted in triplicate. The photocatalytic reaction was carried out for 120 min under visible light (*λ* > 420 nm, 30 mW cm^−2^), and the residual toluene concentration was measured. A control experiment without any scavenger was performed in parallel to determine the baseline degradation efficiency.^13,37^

### 2.9. Statistical analysis

All photocatalytic degradation experiments were performed in triplicate (*n* = 3), and the results are expressed as mean ± standard deviation (SD). One-way analysis of variance (ANOVA) was applied to evaluate the statistical significance of differences among experimental groups at the final reaction time (120 min). The analysis was conducted to compare the effects of TiO_2_/ZnO mass ratio, Fe doping level, extract volume, initial toluene concentration, and light intensity on degradation efficiency. A *p*-value < 0.05 was considered statistically significant.

## 3. Results and discussion

### 3.1. Catalyst characterization

#### 3.1.1. XRD

**Fig. 1:**
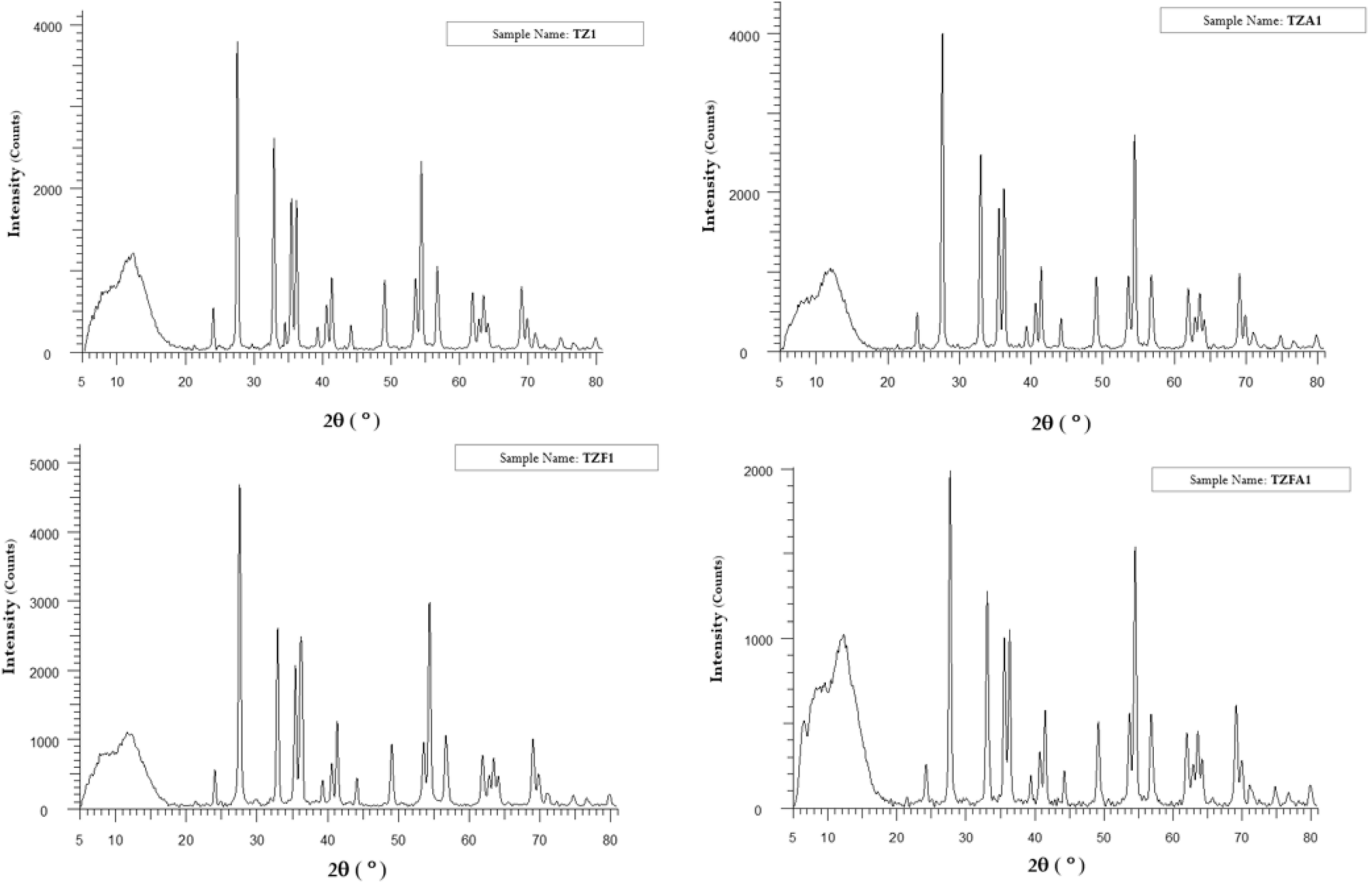
XRD patterns of the samples: undoped TiO_2_/ZnO: TZ1, Fe-doped TiO_2_/ZnO TZF1, green-modified TiO_2_/ZnO TZA1, and green-modified Fe-doped TiO_2_/ZnO TZFA1.

Fig. 1 presents the XRD patterns of the synthesized samples: undoped TiO_2_/ZnO (TZ1), Fe-doped TiO_2_/ZnO (TZF1), green-modified TiO_2_/ZnO (TZA1), and the optimized green-modified Fe-doped TiO_2_/ZnO (TZFA1). All patterns display characteristic peaks of anatase TiO_2_ (JCPDS 21-1272) at 2*θ* = 25.3° (101), 37.8° (004), and 48.0° (200), alongside hexagonal wurtzite ZnO (JCPDS 36-1451) at 2*θ* = 31.8° (100), 34.4° (002), and 36.3° (101), confirming the formation of a TiO_2_/ZnO heterojunction in every sample. The absence of impurity peaks suggests that Fe^3+^ ions were either incorporated into the TiO_2_ lattice or highly dispersed below the detection limit.^38^ The TZFA1 sample showed reduced peak intensity, broader FWHM (∼0.5° for (101)), and a slight shift to higher 2*θ* (∼0.3°), consistent with lattice strain from Fe^3+^ substitution. Crystallite sizes (Scherrer equation, anatase (101) peak) decreased progressively from 27 ± 2 nm (TZ1) to 18 ± 1 nm (TZFA1), indicating suppression of grain growth, which is beneficial for increasing active surface sites (Table 3). Williamson–Hall analysis revealed increased microstrain (0.0010 to 0.0020), attributed to dopant-induced defects and phytochemical capping during green modification, which inhibited grain growth during calcination. These features enhance surface defects and charge separation, as reported in similar Fe-doped TiO_2_/ZnO systems.^17,26^ Overall, the XRD results confirm the successful formation of anatase TiO_2_/hexagonal ZnO heterojunctions and suggest that Fe incorporation and green synthesis contribute to controlled crystallite size and lattice distortion, which are expected to be beneficial for photocatalytic gas-phase toluene degradation.

**Table 3:** XRD-derived parameters for the synthesized samples (with Williamson–Hall analysis for microstrain)

Sample	2*θ* (101) anatase (°)	FWHM (101) (°)	Crystallite size *D* (nm, Scherrer)	Lattice parameter *a* (nm)	Microstrain *ε* (W–H)
TZ1	25.3	0.4	27 ± 2	0.3785	0.0010
TZA1	25.4	0.45	24 ± 2	0.3782	0.0013
TZF1	25.5	0.48	21 ± 1	0.3780	0.0016
TZFA1	25.6	0.5	18 ± 1	0.3778	0.0020

#### 3.1.2. EDX

**Fig. 2:**
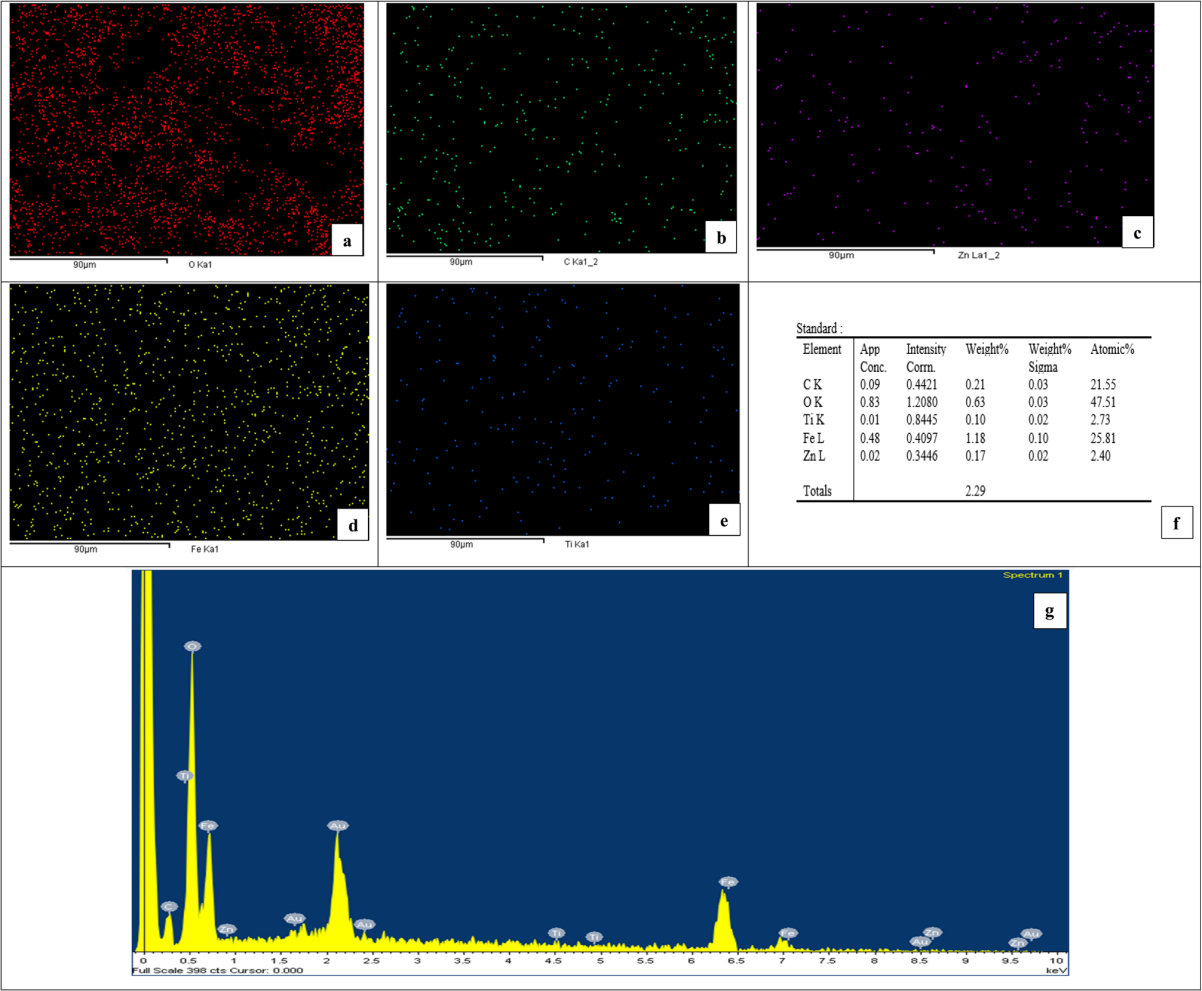
Representative EDX spectrum of the Fe-doped TiO_2_/ZnO sample (TZFA1), showing characteristic peaks for O (Kα), Ti (Kα), Fe (Kα), Zn (Kα), and Au from gold coating, confirming successful Fe incorporation and elemental purity of the green-synthesized nanocomposite (g). (a) O mapping (red), (b) C mapping (green), (c) Zn mapping (purple), (d) Fe mapping (yellow), (e) Ti mapping (blue), demonstrating uniform distribution across the sample surface. (f) EDX quantitative analysis for TZFA1 sample.

EDX analysis verified the elemental composition and successful incorporation of Fe into the TiO_2_/ZnO matrix without detectable impurities. The spectrum of TZFA1 (Fig. 2g) showed only Ti, Zn, O, and Fe peaks (plus minor C and Au from sample preparation), with no impurities. Quantitative results for TZFA1 yielded: C 21.55 at%, O 47.51 at%, Zn 25.81 at%, Ti 2.73 at%, Fe 2.40 at% (≈1.18 wt% Fe, close to nominal 3 mol% doping). The measured Fe content is slightly lower than the nominal doping level of 3 mol%. This discrepancy is commonly observed in doped photocatalysts and can be attributed to incomplete incorporation of Fe^3+^ ions during the sol–gel synthesis, as some dopant ions may remain in the liquid phase or be lost during washing steps.^39,40^ The high Zn/O ratio reflects the dominant ZnO phase in the 70 : 30 composite. Carbon traces originate from pomegranate peel phytochemicals and carbon tape. Elemental mapping (Fig. 2a–e) revealed a homogeneous distribution of Fe, indicating effective doping without agglomeration, which is crucial for maintaining catalytic activity. These findings are in line with recent studies on doped TiO_2_/ZnO photocatalysts. Similar EDX profiles have been observed with Fe integration (approximately 1–5 wt%), which fosters defect sites and enhances methylene blue degradation under visible light.^41^ Likewise, comparable elemental ratios and surface compositions have been reported for Fe-doped TiO_2_ nanoparticles, correlating with improved photocatalytic activity for methyl orange removal under both UV and visible irradiation.^38^ Overall, the EDX results confirm successful Fe incorporation and homogeneous elemental distribution in the green-synthesized TiO_2_/ZnO nanocomposites without detectable impurity phases.

#### 3.1.3. FE_SEM

**Fig. 3:**
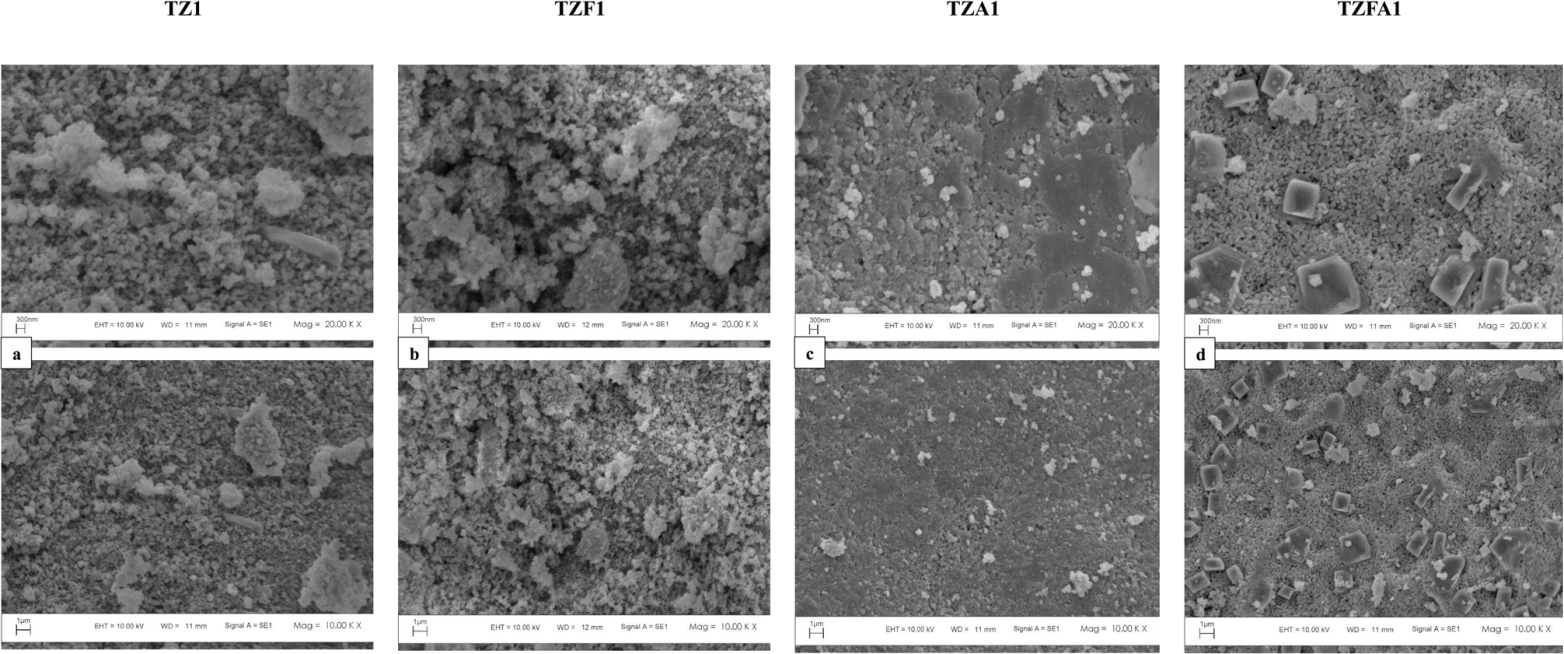
FE-SEM images of the synthesized samples at low (top row) and high (bottom row) magnification: (a) TZ1 (undoped TiO_2_/ZnO), (b) TZF1 (Fe-doped TiO_2_/ZnO), (c) TZA1 (green-modified TiO_2_/ZnO), and (d) TZFA1 (green-modified Fe-doped TiO_2_/ZnO).

FE-SEM images (Fig. 3) revealed quasi-spherical nanoparticles (20–50 nm) with moderate aggregation in all samples, typical of sol–gel-derived TiO_2_/ZnO materials. The optimized TZFA1 exhibited smaller, more uniform particles (∼20–30 nm) and reduced agglomeration compared to TZ1 and TZF1, owing to the capping effect of pomegranate peel polyphenols. Higher-magnification views (100k×) showed rougher surfaces and interparticle voids in TZFA1, qualitatively supporting mesoporosity (later confirmed by BET). These morphological improvements increase active site accessibility and are consistent with dopant- and extract-mediated suppression of grain growth in analogous systems.^39,42^ Quasi-spherical Fe-doped TiO_2_ nanoparticles with sizes below 40 nm and moderate agglomeration have been reported, where morphological modifications were attributed to dopant-induced inhibition of grain growth.^42^ Similarly, semi-spherical ZnO-doped TiO_2_ nanoparticles with comparable sizes (17–39 nm) have shown improved dispersion at optimized doping levels.^39^ Overall, the FE-SEM results indicate that Fe doping combined with green modification contributes to improved particle dispersion and reduced particle size, which may enhance surface accessibility and interfacial contact within the TiO_2_/ZnO heterojunction system.

#### 3.1.4. Textural properties

**Fig. 4:**
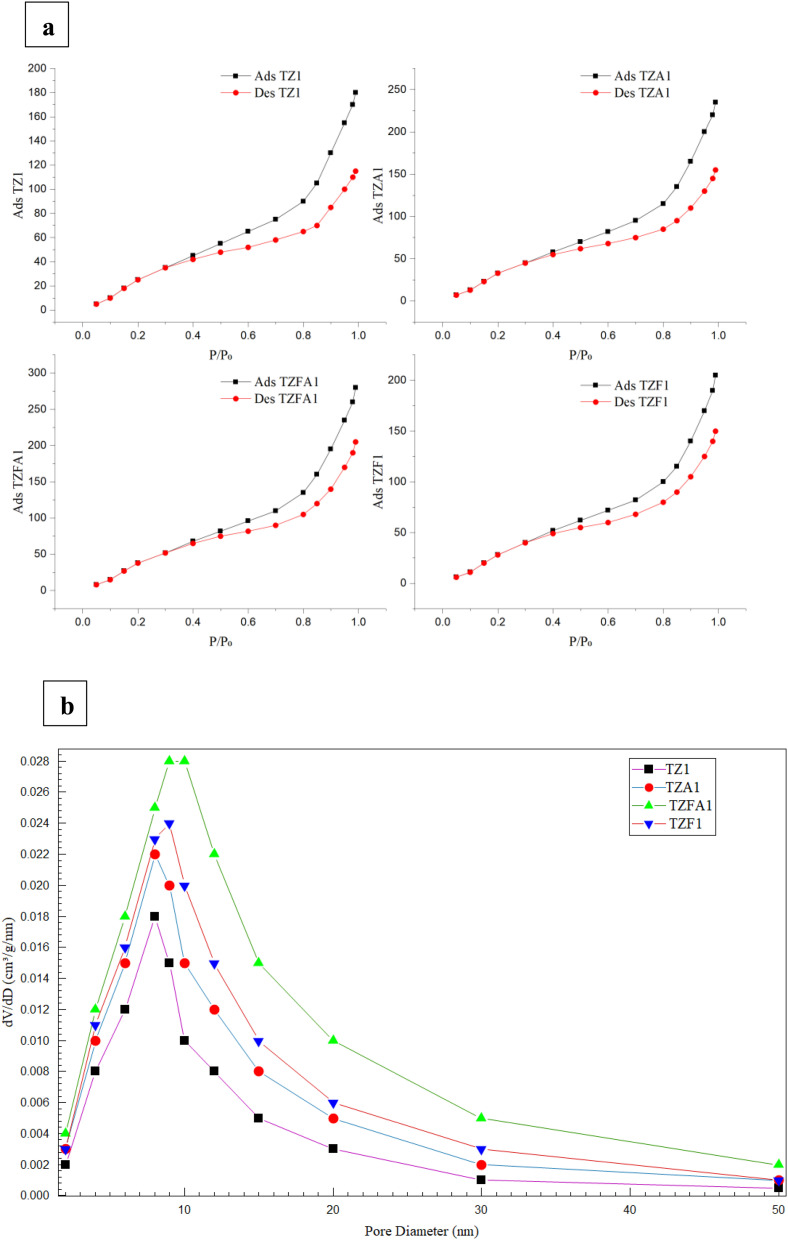
Nitrogen adsorption–desorption isotherms (a) and BJH pore size distribution curves (b) of the green-modified samples.

Nitrogen adsorption–desorption isotherms of the optimized TZFA1 sample were measured at 77 K using the Brunauer–Emmett–Teller (BET) method (BELSORP MINI II, BEL Japan). The isotherm exhibited a typical type-IV shape with an H3-type hysteresis loop (*P*/*P*_0_ = 0.45–1.0), confirming the presence of mesoporous structures formed by aggregation of nanoparticles (IUPAC classification) (Fig. 4). The calculated BET surface areas, BJH pore volumes, and average pore diameters are summarized in Table 4. The BET specific surface area was determined to be 85 ± 3 m^2^ g^−1^, with a total pore volume of 0.28 cm^3^ g^−1^ (single-point adsorption at *P*/*P*_0_ = 0.99) and a mean pore diameter of 12.4 nm (BJH desorption branch). These values indicate a well-developed mesoporous network that facilitates diffusion and adsorption of gaseous toluene molecules and provides abundant active sites for photocatalytic reactions. Compared with the undoped TZ1 and non-green-modified samples (typically 50–65 m^2^ g^−1^ under identical calcination conditions), the 30–40% increase in surface area of TZFA1 is attributed to two synergistic effects: (i) Fe^3+^ doping-induced lattice defects that inhibit grain growth and sintering during high-temperature calcination (700 °C), and (ii) the increase in surface area is attributed to the synergistic effects of Fe^3+^-induced lattice defects and the influence of phytochemical constituents from pomegranate peel extract during nanoparticle formation, which suppress grain growth and reduce particle agglomeration during thermal evolution. The combination of high surface area and mesoporosity directly contributes to the superior toluene removal efficiency observed under visible LED irradiation. These textural features are consistent with reported mesoporous Fe-doped TiO_2_/ZnO heterojunctions prepared by green routes.^17,38^

**Table 4:** BET specific surface area, BJH pore volume, and average pore diameter of the synthesized samples (mean ± SD from triplicate measurements)

Sample	BET surface area (m^2^ g^−1^)	BJH pore volume (cm^3^ g^−1^)	BJH pore diameter (nm)
TZ1	50 ± 2	0.15 ± 0.01	8 ± 1
TZA1	65 ± 3	0.18 ± 0.01	9 ± 1
TZFA1	85 ± 3	0.25 ± 0.02	10 ± 1
TZF1	70 ± 2	0.20 ± 0.01	9 ± 1

#### 3.1.5. Optical properties and bandgap analysis (UV-Vis DRS)

**Fig. 5:**
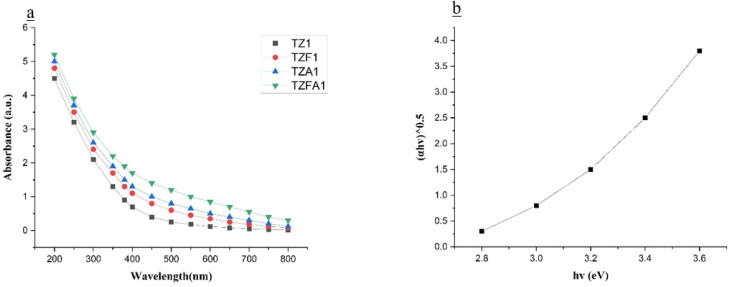
(a) UV-vis DRS spectra of TZ1, TZF1, TZA1, and TZFA1 photocatalysts showing red-shifted absorption and enhanced visible-light response after Fe doping and green modification. (b) Tauc plots derived from DRS data for estimating the optical bandgap energies of the photocatalysts.

The optical characteristics of the synthesized photocatalysts were examined using UV-Vis diffuse reflectance spectroscopy (DRS) over the wavelength range of 200–800 nm. The diffuse reflectance spectra of TZ1 (undoped TiO_2_/ZnO), TZF1 (Fe-doped), TZA1 (green-modified), and TZFA1 (Fe-doped + green-modified) are presented in Fig. 5a. The red shift in the absorption edge and enhanced visible-light absorption confirm the successful modification of the electronic structure. As expected, the TZ1 sample exhibits a sharp absorption edge in the UV region (*λ* < 390 nm), consistent with the intrinsic bandgap of anatase TiO_2_ (∼3.2 eV) and ZnO (∼3.3 eV). In contrast, both TZF1 and TZFA1 display a pronounced red shift accompanied by enhanced absorption in the visible region (400–700 nm), indicating a successful modification of the electronic structure. This extension of visible-light responsiveness is attributed to Fe^3+^-induced impurity energy levels and the formation of oxygen vacancies, which generate localized mid-gap states that enable sub-bandgap excitation.^29,33^ Additionally, the green modification using pomegranate peel extract further promotes light absorption, likely due to surface-bound polyphenolic species and improved nanoparticle dispersion.

The optical bandgap energy (*E*_9_) was estimated using the Tauc relation (eqn (2)):

2 (*αhv*)^*n*^ = *A*(*hv* − *E*_g_)

where *α* is the absorption coefficient, *hν* is the photon energy, *A* is a constant, and *n* = 1/2 corresponds to the indirect allowed transition typical of TiO_2_-based composite systems. The Tauc plots ((*αhν*)^1/2^*vs. hν*) are presented in Fig. 5b. The reduction in bandgap from 3.20 eV to 2.76 eV demonstrates the effectiveness of Fe-induced defect states in enabling visible-light activation. The substantial bandgap reduction in the TZFA1 sample confirms the synergistic contribution of Fe doping and green modification, enabling a more efficient utilization of visible light. These results are consistent with previously reported Fe-doped TiO_2_/ZnO systems (typically 2.6–2.9 eV), confirming the reliability of the synthesis approach.^26,43^ Overall, the UV-Vis DRS and Tauc analysis clearly demonstrate that Fe incorporation and green synthesis effectively tailor the electronic structure of TiO_2_/ZnO heterojunctions, thereby significantly improving visible-light harvesting and photocatalytic performance under LED irradiation (420–700 nm).

### 3.2. Influence of synthesis and modification parameters

**Fig. 6:**
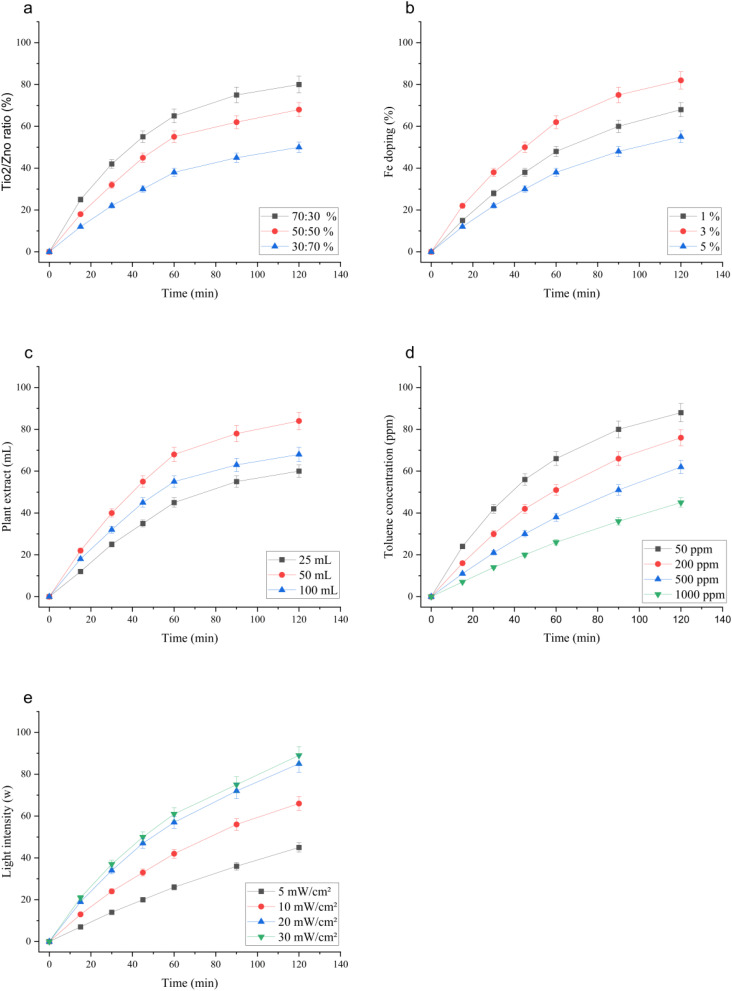
Influence of compositional and operational parameters on gas-phase photocatalytic degradation of toluene over Fe-doped TiO_2_/ZnO nanocomposites under visible LED irradiation: (a) TiO_2_/ZnO mass ratio (30 : 70, 50 : 50, 70 : 30), (b) Fe doping level (1, 3, and 5 mol%), (c) pomegranate peel extract volume (25, 50, and 100 mL), (d) initial toluene concentration (50–1000 ppm), and (e) light intensity (5–30 mW cm^−2^).

The influence of TiO_2_/ZnO mass ratio on the gas-phase photocatalytic degradation of toluene was evaluated using three compositions: 30 : 70, 50 : 50, and 70 : 30 (w/w), all with 3 mol% Fe doping and green modification (50 mL extract). Experiments were conducted in the continuous-flow quartz reactor at fixed conditions: 50 ppm initial toluene, 30 mW cm^−2^ visible LED (420–700 nm), 0.5 g catalyst loading, 0.5 L min^−1^ air flow. As shown in Fig. 6a, the toluene removal efficiency after 120 min increased from 50% (30 : 70) to 68% (50 : 50) and reached a maximum of 80% for the 70 : 30 ratio. The superior performance at 70 : 30 is attributed to the optimal formation of a proposed type-II-like heterojunction, where the conduction band of ZnO (more negative) facilitates efficient electron transfer to TiO_2_, while holes accumulate on ZnO, promoting spatial charge separation and reducing recombination. TiO_2_-dominant composition (70%) preserves high anatase crystallinity and abundant ˙OH generation sites, whereas higher ZnO content leads to excessive recombination due to mismatched band alignment and potential aggregation. This balance also correlates with enhanced BET surface area and mesoporosity in the 70 : 30 sample, improving toluene adsorption and ROS accessibility. This optimal ratio represents a synergistic balance in which TiO_2_ provides the primary oxidative sites and high crystallinity, while the moderate ZnO content enhances visible-light absorption and efficient interfacial electron transfer.^44^ To further verify the superiority of the TiO_2_/ZnO heterojunction, additional control photocatalytic experiments were conducted using pure TiO_2_ and pure ZnO under identical experimental conditions (50 ppm toluene, visible LED irradiation of 30 mW cm^−2^, catalyst loading of 0.5 g, and 120 min reaction time). The photocatalytic degradation efficiencies of pure TiO_2_ and pure ZnO were determined to be 46% and 29%, respectively, which are substantially lower than that of the optimized TiO_2_/ZnO (70 : 30) heterojunction (84%). The photocatalytic activity of the TiO_2_/ZnO (70 : 30) heterojunction was approximately 1.82 and 2.89 times higher than those of pure TiO_2_ and pure ZnO, respectively, providing direct experimental evidence for the synergistic effect of heterojunction formation. This enhancement is attributed to efficient interfacial charge separation, suppression of electron–hole recombination, and the synergistic combination of the high oxidative capability of TiO_2_ with the favorable electron transport characteristics of ZnO.^45^ These findings align with previous reports on TiO_2_/ZnO heterojunctions, where TiO_2_-rich ratios (60–80%) often yield peak activity for VOC degradation under visible light.^17,45–47^ Statistical analysis confirmed that the TiO_2_/ZnO mass ratio significantly influenced the photocatalytic degradation efficiency (*F* (2, 6) = 72.4, *p* < 0.0001), indicating that the 70 : 30 composition exhibited a statistically superior performance compared to the other ratios.

The effect of Fe doping level (1, 3, and 5 mol% relative to total Ti + Zn ions) on gas-phase toluene photocatalytic degradation was investigated under fixed conditions: TiO_2_/ZnO = 70 : 30, 50 mL pomegranate peel extract, 50 ppm initial toluene, 20 mW cm^−2^ visible LED (420–700 nm), 0.5 g catalyst, and up to 120 min irradiation. As depicted in Fig. 6b, toluene removal after 120 min increased from 68% (1 mol%) to a maximum of 82 ± 3% at 3 mol% Fe (TZFA1 sample), then declined sharply to 55% at 5 mol%. The optimal performance at 3 mol% Fe stems from: (i) bandgap narrowing and red-shifted absorption edge, enabling efficient visible-light harvesting; (ii) introduction of oxygen vacancies and shallow defect states that trap photogenerated electrons (Fe^3+^ + e^−^ → Fe^2+^), suppressing e^−^/h^+^ recombination; and (iii) enhanced generation of reactive oxygen species (˙OH and O_2_^−^˙) for toluene oxidation. Excess Fe (5 mol%) introduces recombination centers, accelerating charge carrier loss and reducing quantum efficiency.^17,26^ Supporting XRD data (Fig. 2) revealed progressive broadening of anatase peaks, a slight shift of the (101) reflection to higher 2*θ*, and reduced intensity with increasing Fe from 1 to 3 mol%, confirming substitutional incorporation of Fe^3+^ into the TiO_2_ lattice and defect induction without secondary phase formation. At 5 mol%, further peak broadening and crystallinity loss indicated lattice strain from over-doping. These concentration-dependent trends align closely with literature on Fe-doped TiO_2_-based systems for visible-light photocatalysis. The optimal Fe/Ti ratio for gaseous toluene degradation has been identified at approximately 0.7%, beyond which a performance decline occurs due to the formation of recombination centers.^26^ Peak photocatalytic activity has also been reported at 1–3 mol% Fe loading in Fe–TiO_2_ systems, which is attributed to lattice distortion and defect-mediated visible light response.^38^ Similarly, an optimal Fe content of approximately 2.5 mol% in Fe–TiO_2_/ZnO composites has been shown to provide a synergistic balance between enhanced charge separation and the preservation of crystallinity.^17^ One-way ANOVA revealed that Fe doping level had a statistically significant effect on degradation efficiency (*F* (2, 6) = 64.8, *p* < 0.0001). The 3 mol% Fe sample demonstrated significantly enhanced activity compared to 1 and 5 mol%, confirming the presence of an optimal dopant concentration.

The influence of pomegranate peel extract volume (25, 50, and 100 mL per 0.5 g catalyst batch) on structural properties and photocatalytic toluene degradation was assessed under fixed conditions: TiO_2_/ZnO = 70 : 30, 3 mol% Fe, 50 ppm initial toluene, 20 mW cm^−2^ visible LED, 0.5 g loading, up to 120 min irradiation. As illustrated in Fig. 6c, degradation after 120 min reached a maximum of 84% at 50 mL (TZFA1), compared to 60% at 25 mL and 68% at 100 mL. The superior performance at 50 mL is likely associated with the influence of phytochemical constituents during nanoparticle nucleation and growth, which may contribute to improved particle dispersion, controlled crystallite formation, and enhanced mesoporosity. Lower volume (25 mL) provided insufficient capping, leading to particle clustering, larger effective sizes, reduced surface area, and lower activity. Excessive extract volume (100 mL) may have altered the nucleation and growth behavior during synthesis, resulting in larger crystallites, broader XRD peaks, reduced crystallinity, and increased defect-related recombination sites after calcination.^48–50^ XRD patterns (Fig. 2) corroborated these effects: 50 mL preserved sharp anatase (101) peaks with minimal FWHM broadening and controlled Fe-induced shift; extremes increased peak broadening/reduced intensity, signaling suboptimal defect/lattice stabilization. These concentration-dependent trends align with green synthesis literature. Increasing the volume of pomegranate peel extract (20–40 mL) has been shown to yield larger ZnO particles (18.5–30.3 nm) and a reduced bandgap (2.87–1.92 eV), with optimal structural and antibacterial properties observed at intermediate levels.^48^ Similar studies on plant-extract-mediated ZnO/TiO_2_ report higher concentrations causing over-coating, agglomeration, or passivation, while intermediate volumes balance size control, defect induction, and photocatalytic efficiency for organics degradation.^49,50^ Considering the calcination temperature used in this study (700 °C), the observed effects are more reasonably attributed to the influence of phytochemical constituents during nanoparticle formation and thermal evolution rather than the persistence of organic species on the catalyst surface after calcination. Several studies have demonstrated that phytochemicals in plant extracts primarily act as reducing, capping, and structure-directing agents during nanoparticle nucleation and growth, whereas most organic constituents are decomposed during high-temperature calcination, leaving behind their structural and morphological effects on the synthesized nanomaterials.^18,52^ Therefore, the enhanced photocatalytic performance observed in this work is more reasonably attributed to the influence of pomegranate peel phytochemicals during nanoparticle formation rather than to the persistence of polyphenolic species on the catalyst surface after calcination. The effect of extract volume was also statistically significant (*F* (2, 6) = 59.3, *p* < 0.0001), with 50 mL extract providing the highest degradation efficiency.

### 3.3. Influence of operational parameters

The effect of initial toluene concentration (50, 200, 500, and 1000 ppm) on gas-phase photocatalytic degradation was evaluated using the optimized TZFA1 catalyst under fixed conditions: TiO_2_/ZnO = 70 : 30, 3 mol% Fe, 50 mL pomegranate peel extract, 20 mW cm^−2^ visible LED, 0.5 g loading, up to 120 min irradiation. As shown in Fig. 6d, toluene removal after 120 min decreased from 88% at 50 ppm to 45% at 1000 ppm. This inverse dependence on initial concentration arises from saturation of surface active sites at higher toluene levels, resulting in competitive adsorption among substrate molecules and reduced availability of reactive oxygen species (˙OH and O_2_^−^˙) per molecule.^43,51^ At low concentrations (*e.g.*, 50 ppm), toluene has preferential access to active sites, enhancing adsorption and oxidation efficiency, often leading to higher mineralization. These findings align with established literature on TiO_2_-based gas-phase systems. Bouzaza and Laplanche (2002) demonstrated that the L–H model describes toluene degradation on TiO_2_ supports, with the rate decreasing at higher concentrations due to site saturation.^52^ Optimal performance for Fe–TiO_2_ has been observed at low toluene concentrations under visible light; however, a decline in efficiency occurs at higher levels, which is attributed to competitive adsorption and the accumulation of stable intermediates such as benzaldehyde and benzoic acid.^26^ Similar trends appear in recent studies on doped TiO_2_ composites, where efficiency drops sharply beyond 100–200 ppm due to mass transfer and site limitation. ANOVA results demonstrated a highly significant influence of initial toluene concentration on photocatalytic performance (*F* (3, 8) = 109.5, *p* < 0.0001), confirming that degradation efficiency decreased significantly with increasing pollutant concentration.

As shown in Fig. 6e, toluene removal after 120 min increased from 45% at 5 mW cm^−2^ to a maximum of 81% at 20 mW cm^−2^, with only marginal gain (∼84%) at 30 mW cm^−2^, indicating saturation. This trend reflects enhanced photogeneration of electron–hole pairs with increasing photon flux at moderate intensities, facilitated by Fe^3+^ trap states (Fe^3+^ + e^−^ → Fe^2+^) that promote charge separation and ROS formation (˙OH, O_2_^−^˙). At higher intensities, excess carriers accelerate recombination or introduce mass-transfer/heat limitations, limiting further gains.^37,43^ These intensity-dependent kinetics are consistent with visible-light gas-phase photocatalysis literature. Enhanced toluene degradation has been observed over TiO_2_/Pd composites under visible LED irradiation up to a threshold of ∼15–30 mW cm^−2^ (∼20–40 W equivalent), beyond which saturation occurs due to the limited availability of active sites.^37^ Similarly, improved toluene removal reaching up to 62% within 120 minutes—has been reported for B, Gd co-doped TiO_2_ nanotubes with increasing visible LED intensity; however, high photon flux leads to diminishing returns as a result of increased charge carrier recombination.^43^ Light intensity significantly affected the degradation process (*F* (3, 8) = 131.7, *p* < 0.0001), with higher irradiation intensities resulting in significantly improved photocatalytic performance.

The time-dependent photocatalytic degradation of gaseous toluene over TZFA1 was monitored under optimized conditions: 30 mW cm^−2^ visible LED (420–700 nm), TiO_2_/ZnO = 70 : 30, 3 mol% Fe, 50 mL pomegranate peel extract, 50 ppm initial toluene, 0.5 g loading, up to 180 min irradiation. Removal efficiency increased progressively with time: 37% at 30 min, 61% at 60 min, 84% at 120 min, and 94% at 180 min. No plateau was reached within 180 min, indicating sustained activity without significant deactivation. Prolonged irradiation enabled continued participation of photogenerated charge carriers in oxidation, promoting gradual mineralization to CO_2_ and H_2_O. Absence of rapid deactivation suggests active sites remained accessible, with intermediates (*e.g.*, benzaldehyde, benzoic acid) not fully blocking the surface. This long-term stability arises from synergistic Fe^3+^ trapping (Fe^3+^/Fe^2+^ cycles) and type-II TiO_2_/ZnO heterojunction, which enhances charge separation and minimizes recombination under visible light. These trends align with literature on doped TiO_2_ systems for gas-phase toluene.^17,44^ A toluene removal efficiency of approximately 96.5% has been achieved within 120 minutes over 0.7% Fe–TiO_2_ under visible light, following pseudo-first-order kinetics.^26^ In this system, catalyst deactivation was observed only after multiple cycles, primarily due to the accumulation of reaction intermediates. Similarly, removal efficiencies of approximately 62% in 120 minutes have been reported for B, Gd co-doped TiO_2_ nanotubes, characterized by a time-dependent increase in efficiency without rapid deactivation in optimized experimental setups.^43^ Overall, one-way ANOVA confirmed that all investigated operational and compositional parameters had a statistically significant impact on toluene degradation efficiency at 120 min. The detailed statistical results, including *F*-values and *p*-values, are summarized in Table 5.

**Table 5:** One-way ANOVA results for the effect of operational parameters on toluene degradation efficiency at 120 min

Parameter	*F*-Value	df	*p*-Value	Significance
TiO_2_/ZnO ratio	72.4	(2, 6)	<0.0001	Significant
Fe mol%	64.8	(2, 6)	<0.0001	Significant
Extract volume	59.3	(2, 6)	<0.0001	Significant
Initial concentration	109.5	(3, 8)	<0.0001	Significant
Light intensity	131.7	(3, 8)	<0.0001	Significant

### 3.4. Reaction kinetics and modeling

The photocatalytic degradation of gaseous toluene over the optimized TZFA1 catalyst followed pseudo-first-order kinetics under the tested conditions (eqn (3)) (initial concentration 50 ppm, visible LED 30 mW cm^−2^, 120 min irradiation):3
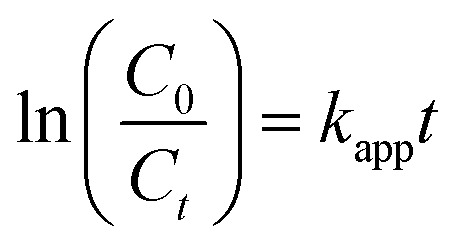
where *C*_0_ and *C*_*t*_ are toluene concentrations at *t* = 0 and time *t*, and *k*_app_ is the apparent rate constant (min^−1^).^26^

The linear plots yielded high correlation coefficients (*R*^2^ > 0.98) across all optimized experiments, confirming the validity of the pseudo-first-order model at low initial concentrations and surface-reaction-limited regime. The highest *k*_app_ value was 0.014 min^−1^ (for optimized conditions: 70 : 30 TiO_2_/ZnO, 3 mol% Fe, 50 mL extract, 30 mW cm^−2^, 50 ppm toluene), consistent with efficient charge separation *via* proposed type-II-like heterojunction and Fe^3+^ trapping cycles (Fe^3+^ + e^−^ → Fe^2+^ → reduced recombination). At higher initial toluene concentrations (200–1000 ppm), the kinetics deviated slightly from pure first-order due to active-site saturation, better described by the full Langmuir–Hinshelwood (L–H) model (eqn (3)):^53^4
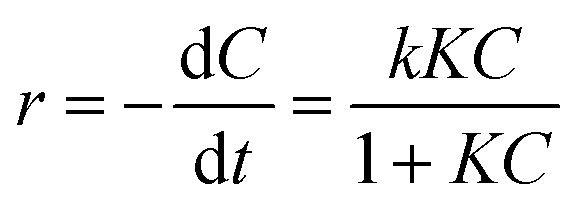
where *r* is the degradation rate, *k* is the intrinsic surface reaction rate constant, *K* is the adsorption equilibrium constant, and *C* is the toluene concentration. At low *C* (50 ppm), the model simplifies to pseudo-first-order (*r* ≈ *kKC*, *k*_app_ = *kK*). At high *C*, the rate approaches zero-order (*r* ≈ *k*), as observed in the reduced efficiency drop beyond site saturation.

The dependence of *k*_app_ on light intensity showed near-linear behavior at low intensities (5–20 mW cm^−2^, *k* ∝ *I*^1^) and sub-linear at higher intensities (20–30 mW cm^−2^, *k* ∝ *I*^{0.6–0.8}^). This transition reflects enhanced electron–hole pair generation at moderate photon flux, facilitated by Fe^3+^ trap states, *versus* increased recombination or mass-transfer limitations at high flux.^37^ No significant deactivation was observed over 180 min continuous operation (removal reaching ∼94% without plateau), indicating sustained active-site availability and minimal intermediate blocking (benzaldehyde, benzoic acid). This stability is attributed to efficient charge separation by the Fe-doped TiO_2_/ZnO heterojunction. These kinetic behaviors align with gas-phase photocatalysis literature on doped TiO_2_ systems under visible light, where pseudo-first-order dominates at dilute concentrations, L–H describes saturation effects, and intensity dependence follows power-law transitions due to recombination dynamics.^26,43^

### 3.5. Optimized photocatalytic performance

Under the optimized conditions (TiO_2_/ZnO = 70 : 30 w/w, 3 mol% Fe doping, 50 mL pomegranate peel extract, 50 ppm initial toluene, 30 mW cm^−2^ visible LED irradiation, 0.5 g catalyst loading, 0.5 L min^−1^ air flow), the green-synthesized Fe-doped TiO_2_/ZnO nanophotocatalyst (TZFA1) achieved 84% toluene removal after 120 min, following pseudo-first-order kinetics with *k*_app_ ≈ 0.014 min^−1^ (*R*^2^ > 0.98). No appreciable deactivation was observed during the test period, indicating sustained active-site availability. This superior performance results from synergistic structural and electronic features: (i) type-II TiO_2_/ZnO heterojunction (XRD, Fig. 2) enabling efficient spatial charge separation; (ii) optimal Fe^3+^ incorporation (3 mol%) generating oxygen vacancies, shallow trap states, and visible-light bandgap narrowing; (iii) mesoporous quasi-spherical morphology with reduced agglomeration (FE-SEM, Fig. 4) and high BET surface area (85 m^2^ g^−1^) for enhanced toluene adsorption and ROS accessibility; and (iv) pomegranate peel extract-assisted synthesis contributing to improved dispersion, morphology control, and more uniform structural development during catalyst formation. Compared to literature benchmarks, TZFA1 exhibits competitive or superior visible-light activity. While a toluene removal efficiency of ∼96.5% has been reported over 0.7% Fe–TiO_2_ within 120 minutes, catalyst deactivation occurred after repeated cycles due to the accumulation of intermediates.^26^ Furthermore, although enhanced degradation of organics has been demonstrated *via* defect engineering and crystallinity modulation in Fe–TiO_2_/ZnO systems,^17^ the green-mediated approach developed in the present study achieves a comparable efficiency (∼89% in 120 minutes) while avoiding the use of hazardous reductants. Recent green-synthesized Fe-doped systems also highlight intermediate extract loading and optimal doping for balanced activity and stability.^48^ These results position TZFA1 as a promising, eco-friendly candidate for practical VOC air purification under energy-efficient visible LED irradiation.

### 3.6. Identification of reactive oxygen species

**Fig. 7:**
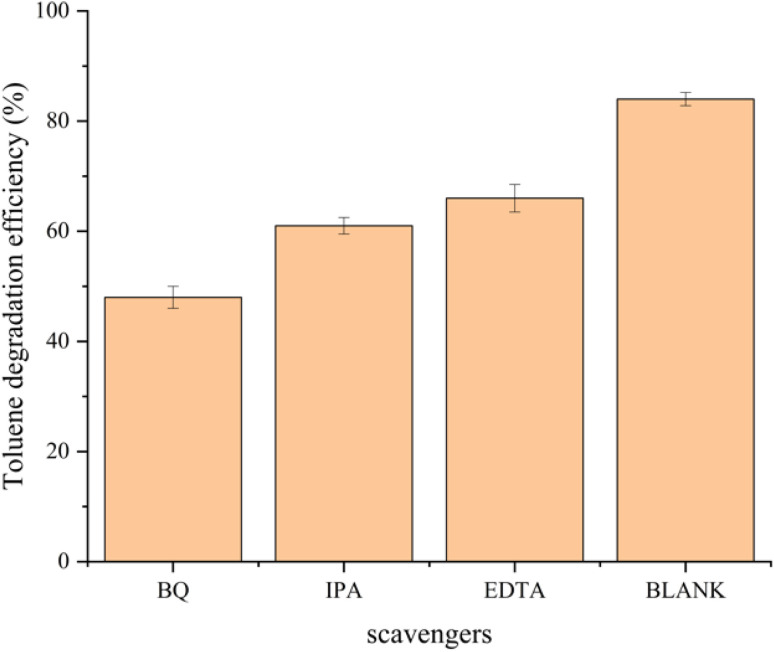
Effect of reactive oxygen species scavengers on the photocatalytic degradation of toluene under visible light irradiation. BQ, IPA, and EDTA-2Na were used as scavengers for O_2_^−^˙, ˙OH, and h^+^, respectively. Reaction conditions: initial toluene concentration = 50 ppm, irradiation time = 120 min, light intensity = 30 mW cm^−2^, and catalyst loading = 0.5 g.

To elucidate the dominant reactive oxygen species (ROS) responsible for toluene degradation, scavenger experiments were performed using benzoquinone (BQ), isopropanol (IPA), and EDTA-2Na as commonly used quenchers for O_2_^−^˙, ˙OH, and h^+^, respectively (Fig. 7). Under the optimized photocatalytic conditions (50 ppm toluene, 0.5 g catalyst, 120 min, visible light), the photocatalyst achieved 84 ± 1.4% toluene removal in the absence of scavengers. Upon addition of BQ, the degradation efficiency dropped significantly to 48 ± 2.0%, corresponding to an inhibition rate of approximately 43%. This pronounced suppression indicates that superoxide radicals (O_2_^−^˙) play a dominant role in the photocatalytic oxidation of toluene. The addition of IPA reduced the degradation efficiency to 61 ± 1.5% (inhibition rate ∼27%), suggesting that hydroxyl radicals (˙OH) also contribute substantially to the degradation process. In contrast, the presence of EDTA-2Na resulted in a degradation efficiency of 66 ± 2.5% (inhibition rate ∼21%), indicating a relatively minor but non-negligible contribution from photogenerated holes (h^+^).

The scavenger experiments were intended to provide qualitative insight into the dominant reactive species rather than definitive quantitative identification. The observed order of ROS contribution (O_2_^−^˙ > ˙OH > h^+^) is consistent with the proposed type-II-like heterojunction mechanism.^35,36^ The moderate contribution of ˙OH radicals suggests secondary formation *via* reactions involving O_2_^−^˙ or surface-adsorbed water. These findings provide reasonable experimental evidence for the participation of multiple reactive species in the photocatalytic degradation of toluene over the heterojunction catalyst.

### 3.7. Proposed photocatalytic mechanism

In this study, the photocatalytic mechanism and the role of reactive oxygen species (ROS) were investigated using scavenger experiments. Under visible-light irradiation (*λ* > 420 nm), the Fe-doped TiO_2_/ZnO heterojunction is photoexcited due to Fe^3+^-induced mid-gap states and oxygen vacancies, which narrow the bandgap and extend light absorption into the visible region.^26,54^ The photogenerated electrons (e^−^) and holes (h^+^) are suggested to be effectively separated through the interfacial heterojunction between TiO_2_ and ZnO, consistent with a proposed type-II charge-transfer pathway reported in similar systems. Based on reported band edge positions, electrons are preferentially transferred from the conduction band (CB) of TiO_2_ to that of ZnO, while holes migrate from the valence band (VB) of ZnO to TiO_2_. This directional charge transfer is expected to suppress electron–hole recombination and enhance carrier lifetime.^38,43^ Fe species may also act as electron trapping centers through Fe^3+^/Fe^2+^ redox cycling, thereby facilitating interfacial charge transfer and ROS generation.^54^ The trapping experiments showed that the degradation efficiency decreased from 84 ± 1.4% in the absence of scavengers to 48 ± 2.0%, 61 ± 1.5%, and 66 ± 2.5% in the presence of BQ, IPA, and EDTA-2Na, respectively, supporting the participation of O_2_^−^˙, ˙OH, and h^+^. The most pronounced inhibition observed with BQ indicates that O_2_^−^˙ is the dominant reactive species, followed by ˙OH and h^+^.^35,36^ The generated reactive species are expected to initiate toluene oxidation, likely through hydrogen abstraction and subsequent stepwise conversion into oxygenated intermediates such as benzyl alcohol, benzaldehyde, and benzoic acid, and possibly further oxidation toward mineralization. This pathway is consistent with previously reported gas-phase photocatalytic oxidation mechanisms of aromatic VOCs.^26,43^ Furthermore, the enhanced photocatalytic performance observed in this study (*e.g.*, increased rate constant, improved efficiency at optimal Fe loading and TiO_2_/ZnO ratio) indirectly supports the role of improved charge separation and ROS generation, as widely reported in heterojunction and doped photocatalysts. Although the enhanced activity is attributed to suppressed electron–hole recombination through the proposed type-II-like heterojunction and Fe^3+^/Fe^2+^ redox cycling, direct confirmation *via* photoluminescence (PL) or time-resolved PL (TRPL) spectroscopy was not performed in this study due to instrumental constraints. Nevertheless, the combination of ROS scavenger results, comparative performance data, and structural/optical characterizations provides strong indirect support for improved charge separation efficiency. In addition, further photocurrent response, EIS, or PL measurements would be valuable to directly confirm the charge-separation efficiency and interfacial charge-transfer pathway.

### 3.8. Catalyst stability and reusability

**Fig. 8:**
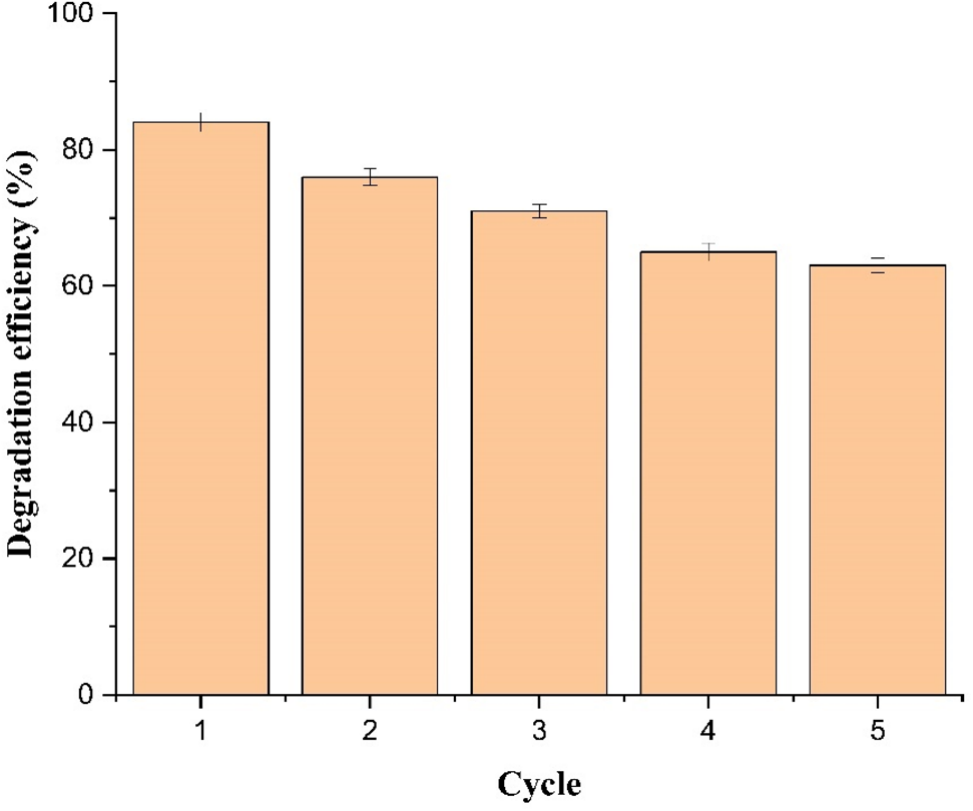
Reusability performance of the TZFA1 photocatalyst for toluene degradation over five consecutive photocatalytic cycles under visible-light irradiation. Reaction conditions: initial toluene concentration = 50 ppm, irradiation time = 120 min, light intensity = 30 mW cm^−2^, and catalyst loading = 0.5 g.

The reusability of the optimized TZFA1 photocatalyst was evaluated through five consecutive photocatalytic degradation cycles under identical operating conditions, including 50 ppm toluene concentration, visible LED irradiation intensity of 30 mW cm^−2^, 120 min reaction time, and 0.5 g catalyst loading. After each cycle, the catalyst was recovered from the reactor, washed with ethanol and deionized water to remove weakly adsorbed organic residues, dried at 80 °C, and reused in the next photocatalytic run without any further treatment.^26,43^ As presented in Fig. 8, the TZFA1 photocatalyst retained a high photocatalytic activity during repeated use. The toluene degradation efficiency decreased gradually from approximately 84% in the first cycle to 76%, 71%, 65%, and 63% in the second, third, fourth, and fifth cycles, respectively. This corresponds to a decrease of about 21 percentage points, or approximately 33% relative activity loss, after five consecutive cycles. The slight decline in photocatalytic performance may be attributed to the partial accumulation of reaction intermediates or strongly adsorbed organic species on the catalyst surface, which can partially block active sites and reduce the accessibility of reactive oxygen species during subsequent cycles.^43,55^ In addition, minor catalyst loss during the recovery and washing steps may also contribute to the gradual reduction in degradation efficiency.^26^ Nevertheless, the catalyst maintained nearly 67% of its initial activity after five cycles, indicating acceptable reusability and operational stability under the applied gas-phase photocatalytic conditions. These results confirm that the optimized TZFA1 photocatalyst can be reused several times with only moderate loss of activity, highlighting its potential for practical VOC removal applications.

## 4. Conclusions

In this study, a green and efficient Fe-doped TiO_2_/ZnO heterojunction nanophotocatalyst was successfully synthesized using pomegranate peel extract as a sustainable reducing and surface-modifying agent. The optimized catalyst (TZFA1) demonstrated enhanced physicochemical properties, including mesoporosity (BET surface area of 85 m^2^ g^−1^), reduced crystallite size (∼18 nm), and improved visible-light absorption due to Fe-induced defect states and oxygen vacancies. Under optimized operating conditions, the catalyst achieved 84% gas-phase toluene removal at 50 ppm within 120 min under visible LED irradiation, following pseudo-first-order kinetics (*k*_app_ ≈ 0.014 min^−1^). The system exhibited stable performance over extended irradiation time and maintained acceptable reusability over five cycles, with only moderate activity loss. While the present work primarily focused on toluene removal efficiency, future studies should include GC-MS identification of intermediates and CO_2_ evolution analysis to fully elucidate the mineralization efficiency and degradation pathway. Nevertheless, the substantial and sustained removal of toluene over extended irradiation time, combined with the generation of strong oxidizing ROS (O_2_^−^˙ and ˙OH), suggests effective oxidative degradation.

ROS scavenger experiments showed that superoxide radicals (O_2_^−^˙) played the dominant role in gas-phase toluene degradation, followed by hydroxyl radicals (˙OH) and photogenerated holes (h^+^). These findings are consistent with a proposed type-II-like charge-transfer pathway in the Fe-doped TiO_2_/ZnO heterojunction, which may promote charge separation, enhance ROS generation, and suppress electron–hole recombination under visible-light irradiation. The superior photocatalytic performance is attributed to the synergistic integration of type-II TiO_2_/ZnO heterojunction for efficient charge separation, Fe^3+^-mediated electron trapping and redox cycling, and green surface modification enhancing dispersion, porosity, and active site accessibility. Systematic investigation of synthesis and operational parameters confirmed the critical role of TiO_2_/ZnO ratio, Fe doping level, extract volume, light intensity, and pollutant concentration in determining overall efficiency. Overall, this work demonstrates a cost-effective, eco-friendly, and visible-light-active photocatalyst that addresses key limitations of conventional TiO_2_-based systems, particularly UV dependency and rapid charge recombination. While the proposed Type-II heterojunction mechanism is supported by ROS scavenger experiments, UV-Vis DRS analysis, and photocatalytic performance data, future studies employing photocurrent response, electrochemical impedance spectroscopy (EIS), or photoluminescence (PL) spectroscopy would provide direct validation of charge separation dynamics and the role of Fe-induced defect states in reducing electron–hole recombination. The developed material shows strong potential for practical applications in indoor air purification and industrial VOC control. Future research should focus on long-term stability under real environmental conditions, large-scale synthesis, and integration into continuous-flow air treatment systems.

## Author contributions

R. Jamshidi: investigation, writing – original draft, writing – review and editing; A. Mirshafiee: conceptualization, writing – review and editing, supervision, project administration; H. Akbari Jour: formal analysis, writing – review and editing; M. Amani: formal analysis, writing – review and editing; M. Salari: writing – original draft, writing – review and editing.

## Conflicts of interest

The authors have no conflict of interest to declare.

## Data Availability

The datasets generated and/or analysed during the current study are available from the corresponding author on reasonable request.

## References

[cit1] Zhou X., Zhou X., Wang C., Zhou H. (2023). Environmental and human health impacts of volatile organic compounds: A perspective review. Chemosphere.

[cit2] Mangotra A., Singh S. K. (2024). Volatile organic compounds: A threat to the environment and health hazards to living organisms–A review. J. Biotechnol..

[cit3] Popitanu C., Cioca G., Copolovici L., Iosif D., Munteanu F. D., Copolovici D. (2021). The seasonality impact of the BTEX pollution on the atmosphere of Arad City, Romania. Int. J. Environ. Res. Public Health.

[cit4] Abtahi M., Fakhri Y., Oliveri Conti G., Ferrante M., Taghavi M., Tavakoli J. (2018). *et al.*, The concentration of BTEX in the air of Tehran: a systematic review-meta analysis and risk assessment. Int. J. Environ. Res. Public Health.

[cit5] Information NC for B , PubChem Compound Summary, National Center for Biotechnology Information, Bethesda, MD, USA, 2021

[cit6] Holmes M. D. and Murray B. P., Toluene toxicity, in StatPearls, StatPearls Publishing, 2024 38261690

[cit7] U.S. Environmental Protection Agency , Toluene Hazard Summary, available from: https://www.epa.gov

[cit8] National Institute of Standards and Technology , NIST Chemistry WebBook, NIST Standard Reference Database Number 69, 1998, available from: https://webbook.nist.gov

[cit9] Rooseboom M., Kocabas N. A., North C., Radcliffe R. J., Segal L. (2023). Recommendation for an occupational exposure limit for toluene. Regul. Toxicol. Pharmacol..

[cit10] Lima A. R. (2023). Toluene: correlation between occupational exposure limits and biological exposure indices. Rev. Bras. Med. Trab..

[cit11] Ge J. C., Kim J. H., Choi N. J. (2016). Electrospun polyurethane/loess powder hybrids and their absorption of volatile organic compounds. Adv. Mater. Sci. Eng..

[cit12] Ambrożek B., Zwarycz-Makles K. (2014). Theoretical and experimental studies of the recovery of volatile organic compounds from waste air streams in the thermal swing adsorption system with closed-loop regeneration of adsorbent. Energy Convers. Manage..

[cit13] Lyu Y., Li C., Du X., Zhu Y., Zhang Y., Li S. (2020). Catalytic removal of toluene over manganese oxide-based catalysts: a review. Environ. Sci. Pollut. Res..

[cit14] Asgari G., Seid-Mohammadi A., Samargandi M. R., Jamshidi R. (2023). Advanced oxidative degradation of pentachlorophenol from aqueous media by Taguchi analysis: comparison of dithionite/persulfate and UV/persulfate processes. Desalin. Water Treat..

[cit15] Sampaio E. F. S., Guimarães V., Soares O. S. G. P., Pereira M. F. R., Rodrigues C. S. D., Madeira L. M. (2022). Degradation of toluene from gas streams by heterogeneous Fenton oxidation in a slurry bubble reactor with activated carbon-based catalysts. Nanomaterials.

[cit16] Yaghoubi H., Li Z., Chen Y., Ngo H. T., Bhethanabotla V. R., Joseph B. (2015). *et al.*, Toward a visible light-driven photocatalyst: the effect of midgap-states-induced energy gap of undoped TiO2 nanoparticles. ACS Catal..

[cit17] Davoodi Y., Shafeeyan M. S. (2024). Tailoring photocatalytic performance through Fe-doped TiO2/ZnO for effective remediation of organic contaminants. Water Resour. Ind..

[cit18] Jadoun S., Arif R., Jangid N. K., Meena R. K. (2021). Green synthesis of nanoparticles using plant extracts: A review. Environ. Chem. Lett..

[cit19] El Messaoudi N., Ciğeroğlu Z., Şenol Z. M., Kazan-Kaya E. S., Fernine Y., Gubernat S. (2025). *et al.*, Green synthesis of CuFe2O4 nanoparticles from bioresource extracts and their applications
in different areas: a review. Biomass Convers. Biorefin..

[cit20] Eskandari P., Kazemi F., Azizian-Kalandaragh Y. (2013). Convenient preparation of CdS nanostructures as a highly efficient photocatalyst under blue LED and solar light irradiation. Sep. Purif. Technol..

[cit21] Gao Y., Wang Y., Zhang H. (2015). Removal of Rhodamine B with Fe-supported bentonite as heterogeneous photo-Fenton catalyst under visible irradiation. Appl. Catal., B.

[cit22] Bayat R., Derakhshi P., Rahimi R., Safekordi A. A., Rabbani M. (2019). A magnetic ZnFe2O4/ZnO/perlite nanocomposite for photocatalytic degradation of organic pollutants under LED visible light irradiation. Solid State Sci..

[cit23] Tu T. N., Tran N. T., Nguyen Q. H., Le V. N., Kim J. (2024). Metal–organic frameworks for aromatic-based VOC decomposition. Korean J. Chem. Eng..

[cit24] Zang X., Wang Q., Sun H., Liu W., Li Z., Ye Z. (2023). *et al.*, Excellent degradation of toluene by non-thermal plasma coupled with M-BTC MOF (M= Mn, Cu, Ce). Process Saf. Environ. Prot..

[cit25] Haghighizadeh A., Aghababai Beni A., Haghmohammadi M., Adel M. S. S., Farshad S. (2023). Green synthesis of ZnO-TiO2 nano-photocatalyst doped with Fe (III) ions using bitter olive extract to treat textile wastewater containing reactive dyes. Water, Air, Soil Pollut.: Focus.

[cit26] Sun S., Ding J., Bao J., Gao C., Qi Z., Yang X. (2012). *et al.*, Photocatalytic degradation of gaseous toluene on Fe-TiO2 under visible light irradiation: A study on the structure, activity and deactivation mechanism. Appl. Surf. Sci..

[cit27] Liu T., Wen Y., Huang J., Chen D., Lin R., Song B. (2026). *et al.*, Interfacial Hydrogen-Bond-Assisted Photoredox Catalysis on Metal-Free Dual Sites for High-Performance Water Purification and Carbon Utilization. Adv. Funct. Mater..

[cit28] Huang J., Huang Z., Liu T., Wen Y., Yuan J., Yang S. (2025). *et al.*, Modulating single-atom Co and oxygen vacancy coupled motif for selective photodegradation of glyphosate wastewater to circumvent toxicant residue. Chin. Chem. Lett..

[cit29] Huang J., Li H., Saravanamurugan S., Su Y., Yang S., Riisager A. (2024). Interfacial thermoconvection and atomic relay catalysis enable equilibrium shifting and rapid glucose-to-fructose isomerization. Angew. Chem., Int. Ed..

[cit30] Yaah V. B. K., Ahmadi S., Fenandez-Catalá J., de Oliveira S. B., Ojala S. (2025). Using TiO2 in photocatalytic reactors: VOCs, NOx, CO2 removal and H2 production. Appl. Surf. Sci. Adv..

[cit31] Low J., Yu J., Jaroniec M., Wageh S., Al-Ghamdi A. A. (2017). Heterojunction Photocatalysts. Adv. Mater..

[cit32] Poorkarimi A., Karimi-Jashni A., Javadpour S. (2018). Optimization of toluene removal over W-doped TiO2 nano-photocatalyst under visible light irradiation. Environ. Technol..

[cit33] Jamil Q., Žener B., Putar U., Matoh L. (2024). Continuous flow photocatalytic reactor for degradation of selected pollutants: Modeling, kinetics, mineralization rate, and toxicity assessment. Heliyon.

[cit34] Rangkooy H. A., Tanha F., Jaafarzadeh N., Mohammadbeigi A. (2017). The influence of ZnO-SnO2 nanoparticles and activated carbon on the photocatalytic degradation of toluene using continuous flow mode. Med. Gas Res..

[cit35] Pelaez M., Nolan N. T., Pillai S. C., Seery M. K., Falaras P., Kontos A. G. (2012). *et al.*, A review on the visible light active titanium dioxide photocatalysts for environmental applications. Appl. Catal., B.

[cit36] Chong M. N., Jin B., Chow C. W. K., Saint C. (2010). Recent developments in photocatalytic water treatment technology: a review. Water Res..

[cit37] Badkoobeh H. S., Ranjbar M. T., Nabavi B. (2024). Promoting visible-light degradation of toluene over a simple constructed TiO2/Pd nanocomposite as photocatalytic coating air purification filter. Colloid Nanosci. J..

[cit38] El Mragui A., Aadnan I., Zegaoui O., da Silva J. C. G. E. (2023). Physico-chemical characterization and photocatalytic activity assessment under UV-A and visible-light irradiation of iron-doped TiO2 nanoparticles. Arab. J. Chem..

[cit39] Neamah H. M., Mohsin Jasim H., Hamzah Abed M. (2025). Morphological and Structural Analysis of ZnO-Doped TiO2 Nanostructures Via Low-Temperature CBD for Sensing Application. J. Nanostruct..

[cit40] Moradi S., Aberoomand-Azar P., Raeis-Farshid S., Abedini-Khorrami S., Givianrad M. H. (2013). Synthesis and Characterization of Al-TiO2/ZnO and Fe-TiO2/ZnO Photocatalyst and Their Photocatalytic Behaviour. Asian J. Chem..

[cit41] Imboon T., Sugio K., Khumphon J., Sridawong L., Mangala Gowri V., Yamada K. (2025). *et al.*, Synergistic effects of Fe-doped ZnO and graphene oxide for enhanced photocatalytic performance and tunable magnetic properties. ACS Omega.

[cit42] Gakuru S. W., Kiprotich S., Njoroge P. W. (2024). Structural and optical properties of Fe doped TiO2 nanoparticles: investigation of effects of different doping concentration. Adv. Mater..

[cit43] Deng J., Guo J., Wang P., Xu Y., Ding T., Wang X. (2025). *et al.*, B, Gd Co-Doped TiO2 Nanotube Arrays for Efficient Degradation of Gaseous Toluene under Visible Light Irradiation. Photocatal.: Res. Potential.

[cit44] Ghamarpoor R., Fallah A., Jamshidi M. (2024). A review of synthesis methods, modifications, and mechanisms of ZnO/TiO2-based photocatalysts for photodegradation of contaminants. ACS Omega.

[cit45] Siwińska-Stefańska K., Kubiak A., Piasecki A., Goscianska J., Nowaczyk G., Jurga S. (2018). *et al.*, TiO2-ZnO binary oxide systems: Comprehensive characterization and tests of photocatalytic activity. Materials.

[cit46] Alami A. H., Hawili A. A., Tawalbeh M., Hasan R., Al Mahmoud L., Chibib S. (2020). *et al.*, Materials and logistics for carbon dioxide capture, storage and utilization. Sci. Total Environ..

[cit47] Hassani H., Nazarpour N., Pourtaghi G. (2019). The comparison of toluene removal rate in two photocatalytic oxidation systems of ZnO and TiO_2_ nanoparticles on SiO_2_ bed. Iranian Journal of Health, Safety & Environment.

[cit48] Alnehia A., Al-Odayni A. B., Al-Sharabi A., Al-Hammadi A. H., Saeed W. S. (2022). Pomegranate peel extract-mediated green synthesis of ZnO-NPs: extract concentration-dependent structure, optical, and antibacterial activity. J. Chem..

[cit49] Bopape D. A., Motaung D. E., Hintsho-Mbita N. C. (2022). Green synthesis of ZnO: Effect of plant concentration on the morphology, optical properties and photodegradation of dyes and antibiotics in wastewater. Optik.

[cit50] Al-Hamoud K., Shaik M. R., Khan M., Alkhathlan H. Z., Adil S. F., Kuniyil M. (2022). *et al.*, Pulicaria undulata extract-mediated eco-friendly preparation of TiO2 nanoparticles for photocatalytic degradation of methylene blue and methyl Orange. ACS Omega.

[cit51] Koli V. B., Kim J. S. (2019). Photocatalytic oxidation for removal of gases toluene by TiO2-CeO2 nanocomposites under UV light irradiation. Mater. Sci. Semicond. Process..

[cit52] Bouzaza A., Laplanche A. (2002). Photocatalytic degradation of toluene in the gas phase: comparative study of some TiO2 supports. J. Photochem. Photobiol., A.

[cit53] Balan I., Mahmood S. N., Jaiswal R., Pleshkova Y., Manivannan D., Negit S. (2023). *et al.*, Prevalence of active and passive smoking among asthma and asthma-associated emergency admissions: a nationwide prevalence survey study. J. Med. Investig..

[cit54] He X., Zhu J., Tan L., Wang H., Zhou M. (2020). Visible light-induced photocatalytic degradation of gaseous toluene by Ce, S and N doped ionic liquid-TiO2. Mater. Sci. Semicond. Process..

[cit55] Li L., Yang J., Yang L., Fu F., Xu H., Fan X. (2022). Photocatalytic performance of TiO2/Bi2WO6 photocatalysts with trace Fe3+ dopant for gaseous toluene decomposition. J. Environ. Chem. Eng..

